# YTHDC2 serves a distinct late role in spermatocytes during germ cell differentiation

**DOI:** 10.1101/2023.01.23.525146

**Published:** 2023-01-23

**Authors:** Alexis S. Bailey, Margaret T. Fuller

**Affiliations:** 1 Department of Developmental Biology, Stanford University School of Medicine, Stanford, CA, 94305, USA

## Abstract

Post-transcriptional regulation of gene expression by RNA-binding proteins helps facilitate fast, clean transitions from one cell state to the next during germ cell differentiation. Previously we showed that the RNA helicase YTHDC2 is required for germ cells to properly switch from mitosis to meiosis (Bailey et al., 2017). While YTHDC2 protein is first expressed as male germ cells enter meiosis, when it is needed to shut down the mitotic program, YTHDC2 expression continues to increase and reaches its highest levels later in meiotic prophase, in pachytene spermatocytes. Here we show that YTHDC2 has an additional essential role regulating meiotic progression in late spermatocytes during mouse germ cell differentiation. Inducing conditional knockout of *Ythdc2* during the first wave of spermatogenesis, after the germ cells have already initiated meiotic prophase, allowed *Ythdc2*-deficient germ cells to successfully reach the pachytene stage and properly express many meiotic markers. However, instead of continuing through meiotic prophase and initiating the meiotic divisions, late pachytene spermatocytes failed to transition to the diplotene stage and quickly died. Loss of function of *Ythdc2* in spermatocytes resulted in changes in transcript levels for a number of genes, some up-regulated and some down-regulated, compared to control mid-stage spermatocytes. YTHDC2 interacts with different proteins in early and late spermatocytes, with many of the interacting proteins involved in post-transcriptional RNA regulation and present in RNA granules, similar to YTHDC2. Our findings suggest that YTHDC2 facilitates proper progression of germ cells through multiple steps of meiosis, potentially via several mechanisms of post-transcriptional RNA regulation.

## INTRODUCTION

RNA-binding proteins and post-transcriptional regulation of RNAs play fundamental roles in germ cell development, allowing a properly timed transition from the mitotic to the meiotic program and clean progression of germ cells through meiotic prophase and terminal differentiation. The DExH-box RNA helicase, YTHDC2, is the mammalian homolog of *Drosophila* Benign gonial cell neoplasm (Bgcn), which along with its protein binding partners Bag of marbles (Bam) and Tumorous testis (Tut), regulate the switch from mitotic divisions to meiosis in males through direct interaction with the 3ʹUTR of RNAs in the *Drosophila* germline (Chen et al., 2014; Insco et al., 2012). Previous studies from our lab and others have shown that YTHDC2 is first expressed in early spermatocytes, and is required to properly execute the transition from mitosis to meiosis in the mouse germline (Bailey et al., 2017; Hsu et al., 2017; Jain et al., 2018; Liu et al., 2021; Wojtas et al., 2017). *Ythdc2* null mutant germ cells enter meiotic prophase and start to express some meiotic factors, but fail to down-regulate certain mitotic regulators and have a mixed identity (Bailey et al., 2017; Li et al., 2022). Meiotic germ cells in *Ythdc2* null mutants attempt an aberrant mitosis-like metaphase then quickly die by apoptosis. As YTHDC2 localizes to the cytoplasm and has been shown to directly bind specific RNA transcripts, including the mitotic cyclin *Ccna2* (Bailey et al., 2017; Saito et al., 2022), YTHDC2 likely regulates gene expression post-transcriptionally, similar to *Drosophila Bgcn*.

YTHDC2 protein contains two ankyrin (ANK) repeats, which typically mediate protein-protein interactions (Li et al., 2006). Previous studies have shown that YTHDC2 binds the meiotic protein MEIOC (Abby et al., 2016; Bailey et al., 2017; Li et al., 2022; Soh et al., 2017) as well as the RNA-binding protein RBM46 (Li et al., 2022; Qian et al., 2022). Binding of YTHDC2 to both MEIOC and RBM46 does not require RNA. *Meioc* and *Rbm46* knockout males show the same early spermatocyte death phenotype as *Ythdc2* null mutants (Abby et al., 2016; Peart et al., 2022; Qian et al., 2022; Soh et al., 2017), suggesting that YTHDC2, MEIOC and RBM46 function together to regulate the switch from mitosis to meiosis in males. Directly tying YTHDC2 target RNAs to degradation machinery and supporting a functional role for YTHDC2 in regulating RNA stability, previous studies have also shown that the cytoplasmic 5ʹ → 3ʹ exoribonuclease XRN1 is a direct, RNA-independent binding partner of YTHDC2, interacting via the YTHDC2 ANK domain (Kretschmer et al., 2018; Li et al., 2022; Wojtas et al., 2017).

While YTHDC2 protein expression starts as male germ cells enter meiosis, western blot analysis indicated that YTHDC2 protein levels increase during the first wave of spermatogenesis as germ cells proceed through meiotic prophase (Bailey et al., 2017). As *Ythdc2* null mutant germ cells die very soon after entry into meiotic prophase, it was not possible to investigate whether YTHDC2 plays important roles in male germ cell differentiation after its role in shutting down the mitotic program. Also, the abnormally low levels of many meiotic RNAs such as *Spo11*, observed in testes from *Ythdc2* null mutant males, could be a secondary effect of the death of young spermatocytes early in meiotic prophase. Here we examine the effects of loss of function of *Ythdc2* in later spermatocytes to shed light on another molecular role of this pivotal protein in germ cell development.

Our results show that in addition to its role in the mitosis-to-meiosis transition, YTHDC2 has another critical role in promoting progression of spermatocytes through late meiotic prophase. To carefully study the direct consequences of *Ythdc2* loss of function in late meiotic prophase, we induced conditional knockout of *Ythdc2* at a specific stage during the first wave of spermatogenesis. This strategy allowed us to largely bypass the early mitosis-to-meiosis defects previously observed in *Ythdc2* null mutants. Induced deletion of *Ythdc2* in early spermatocytes allowed the germ cells to properly progress to the pachytene stage and express meiotic markers. However, changes in gene expression were apparent in pachytene stage spermatocytes three days post tamoxifen, followed by elimination of late pachytene spermatocytes by apoptosis prior to the meiotic divisions. Immunoprecipitation experiments revealed that YTHDC2 protein interacts with several RNA-binding proteins with roles in mRNA metabolism in early and late spermatocytes, supporting the idea that YTHDC2 functions at multiple stages of germ cell development to facilitate proper entry and execution of meiosis.

## RESULTS

### YTHDC2 is highly expressed in late spermatocytes.

**Figure 1.**
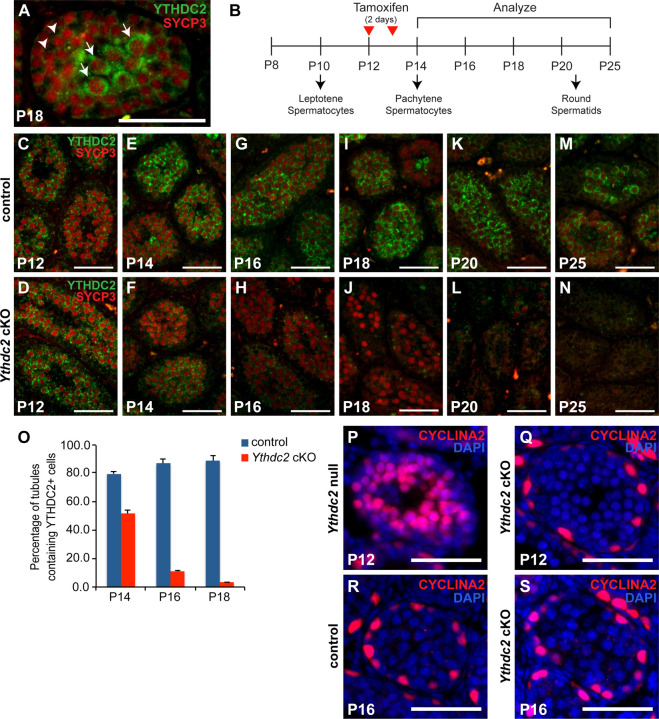
Knockout of *Ythdc2* in late spermatocytes during the first wave of spermatogenesis. (A) Immunofluorescence image of a P18 wild-type mouse testis tubule cross-section stained for YTHDC2 (green) and SYCP3 (red). Arrowheads: leptotene spermatocytes from the second wave of spermatogenesis. Arrows: pachytene spermatocytes from the first wave of spermatogenesis within the lumen of the testis tubule. (B) Diagram of the appearance of germ cell stages during the first wave of spermatogenesis and the timing of tamoxifen injections and analysis of control and *Ythdc2* cKO mice. (C-N) Immunofluorescence images of control (*+/+; Ythdc2*^*flox/∆*^, top) and *Ythdc2* cKO (*UBC-CreERT2; Ythdc2*^*flox/∆*^, bottom) seminiferous tubule cross-sections from (C, D) P12, (E, F) P14, (G, H) P16, (I, J) P18, (K, L) P20 and (M, N) P25 testes stained for YTHDC2 (green) and SYCP3 (red). (O) Percentage of tubules containing YTHDC2-positive cells in P14, P16 and P18 control (blue) and *Ythdc2* cKO (red) testes (n = 2–4 mice per group; t test, P14: p-value = 0.0003, P16: p-value < 0.0001, P18: p-value = 0.0019). Error bars: SEM. (P-S) Immunofluorescence images of testis tubules from (P) P12 *Ythdc2* null (Q) P12 *Ythdc2* cKO (R) P16 control and (S) P16 *Ythdc2* cKO mice stained for CYCLINA2 (red) and DAPI (blue). Scale bars: 50 µm. See also Figure 1-figure supplement 1.

Immunofluorescence staining detected YTHDC2 protein starting in early spermatocytes, with expression continuing throughout meiotic prophase, as previously reported (Bailey et al., 2017; Jain et al., 2018). Similar immunofluorescence staining of cross-sections of testis tubules from males 18 days after birth (P18) showed low levels of YTHDC2 signal in the cytoplasm of early leptotene spermatocytes (Figure 1A, white arrowheads) and high levels of cytoplasmic YTHDC2 signal in the late pachytene spermatocytes that occupy the center of the testis tubules (Figure 1A, white arrows), raising the question of whether YTHDC2 has a critical function in late spermatocytes in addition to its role in early meiotic prophase.

To bypass the early *Ythdc2* null mutant phenotype and examine the function of YTHDC2 in late meiotic prophase, we generated mice bearing a conditional *Ythdc2* allele (*Ythdc2*^*flox*^) (Figure 1-figure supplement 1) with *loxP* sites flanking exons 6 and 7. *Ythdc2*^*flox*^ mice were crossed to our previously generated mice carrying the *Ythdc2* null allele (*Ythdc2*^*∆*^) (Bailey et al., 2017). We then introduced the tamoxifen-inducible transgene *UBC-Cre-ERT2* (Ruzankina et al., 2007), which expresses Cre-ERT2 under the control of the ubiquitin C (UBC) promoter. Treatment of *UBC-Cre-ERT2; Ythdc2*^*flox/∆*^ (*Ythdc2* cKO) juvenile mice with tamoxifen at P12 and P13, when the first wave of germ cells have already entered meiotic prophase and are in the leptotene/zygotene stage (Figure 1B), allowed expression of YTHDC2 protein in early-stage spermatocytes but substantially reduced levels of YTHDC2 protein expression in late-stage spermatocytes. YTHDC2 protein was expressed at similar levels in control and *UBC-Cre-ERT2; Ythdc2*^*flox/∆*^ early spermatocytes at P12, the time of the first tamoxifen injection (Figure 1C,D). Immunofluorescence staining showed that YTHDC2 protein levels started to decrease by just one day after the second tamoxifen injection (P14) in *Ythdc2* cKO tubules compared to controls (Figure 1F vs. E), although low levels of YTHDC2 protein were still detected in approximately 50% of the *Ythdc2* cKO tubules (Figure 1O). By P16, while control spermatocytes continued to have high levels of YTHDC2 protein expression (Figure 1G), the level of YTHDC2 protein in spermatocytes detected by immunofluorescence staining was considerably lower in *Ythdc2* cKO testis tubules (Figure 1H). Quantification of the percentage of tubules that contained YTHDC2-positive spermatocytes confirmed that YTHDC2 protein expression was below the level of detection by our immunofluorescence staining in the majority of *Ythdc2* cKO testis tubules by P16 (Figure 1O).

### YTHDC2 is required for late spermatocytes to progress through meiotic prophase.

**Figure 2.**
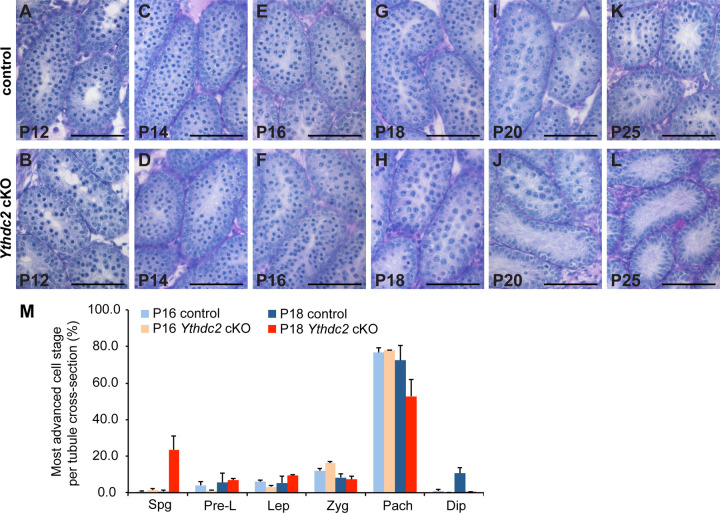
YTHDC2 is required for late spermatocytes to progress through meiotic prophase. (A-L) Sections of mouse seminiferous tubules stained with periodic acid-Schiff (PAS) from (A, B) P12, (C, D) P14, (E, F) P16, (G, H) P18, (I, J) P20 and (K, L) P25 control (*+/+; Ythdc2*^*flox/∆*^, top) and *Ythdc2* cKO (*UBC-CreERT2; Ythdc2*^*flox/∆*^, bottom) testis. Scale bars: 50 µm. (M) Percentage of tubule cross-sections containing spermatogonia (Spg), preleptotene (Pre-L), leptotene (Lep), zygotene (Zyg), pachytene (Pach) or diplotene (Dip) as the most advanced cell stage in P16 control (light blue), P16 *Ythdc2* cKO (light orange), P18 control (blue) and P18 *Ythdc2* cKO (red) testes (n = 2 mice per group; > 115 tubules counted per cross-section). Error bars: SEM.

Analysis of testis tubule cross-sections from the *Ythdc2* cKO males showed that the timing of the tamoxifen treatment allowed male germ cells to proceed past the initial requirement for YTHDC2 function early in meiotic prophase. Our previous studies revealed that in *Ythdc2* null mutant mice, a large number of germ cells underwent abnormal chromosome condensation soon after initiating meiotic prophase (Bailey et al., 2017), visible as abnormal metaphase-like spermatocytes in P12 testes. This phenotype was also observed in *Meioc* and *Rbm46* knockout males (Abby et al., 2016; Qian et al., 2022; Soh et al., 2017). Quantification of testis tubule cross-sections revealed that only a small percentage of tubules in *Ythdc2* cKO mice contained cells with condensed chromosomes. This observation was made at both P16 (control 1.4% vs. *Ythdc2* cKO 8.7%) and P18 (control 0% vs. *Ythdc2* cKO 3.9%). Consistent with the small number of cells with abnormal chromosomal condensation in *Ythdc2* cKO mice, *Ythdc2* cKO spermatocytes did not express the abnormal high levels of CYCLINA2 in spermatocytes previously observed in P12 *Ythdc2* null mutant males (Figure 1Q,S vs. P), indicating that inducing knockout of *Ythdc2* starting at P12 allowed the majority of germ cells to get past the requirement to shut down the mitotic program during the first wave of spermatogenesis. Examination of histological sections of P16 testes showed that unlike *Ythdc2* null mutant germ cells, which never reached the pachytene spermatocyte stage (Bailey et al., 2017), *Ythdc2* cKO germ cells moved successfully through the early spermatocyte stages and reached the pachytene stage at a similar frequency to controls (Figure 2E,F and M). By P20, while control tubules were filled with late-stage spermatocytes (Figure 2I), the majority of tubules in *Ythdc2* cKO mice lacked late spermatocytes (Figure 2J). *Ythdc2* cKO tubules were empty of all spermatocyte stages by P25 (Figure 2L).

**Figure 3.**
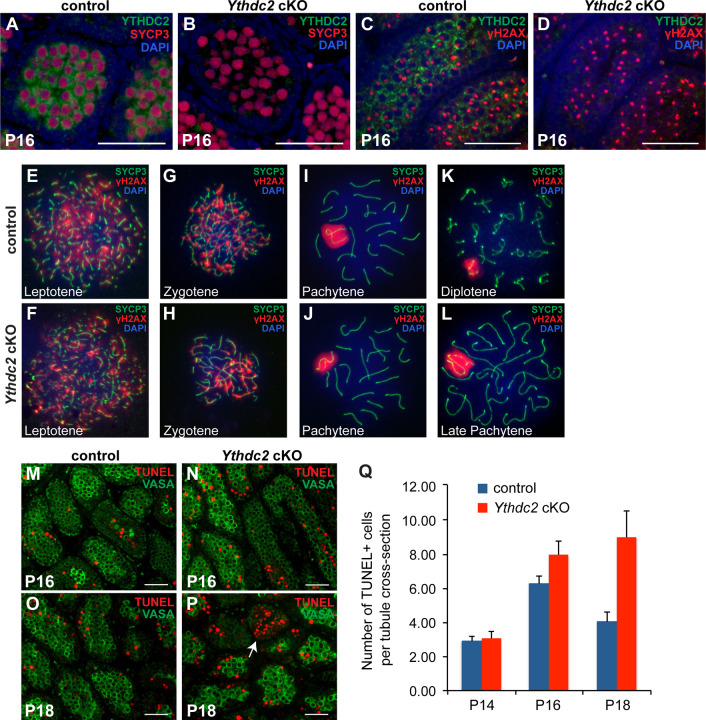
Spermatocytes lacking YTHDC2 reach the pachytene stage but then quickly die. (A, B) Testis from P16 (A) control (*+/+; Ythdc2*^*flox/∆*^) and (B) *Ythdc2* cKO (*UBC-CreERT2; Ythdc2*^*flox/∆*^) mice stained for YTHDC2 (green), SYCP3 (red) and DAPI (blue). (C, D) Testis from P16 (C) control and (D) *Ythdc2* cKO mice stained for YTHDC2 (green), γH2AX (red) and DAPI (blue). (E-L) Immunofluorescence images of germ cell spreads from P18 control (top) and *Ythdc2* cKO (bottom) mice stained for SYCP3 (green), γH2AX (red) and DAPI (blue). (M-P) Testis tubule cross-sections from (M, N) P16 and (O, P) P18 control and *Ythdc2* cKO mice labeled with TUNEL (red) and VASA (green). Arrow: Tubule filled with TUNEL-positive cells. Scale bars: 50 µm. (Q) Number of TUNEL-positive cells per tubule cross-section in P14, P16 and P18 control (blue) and *Ythdc2* cKO (red) testes (n = 2–3 mice per group). Error bars: SEM. See also Figure 3-figure supplement 1 and Figure 3-figure supplement 2.

Immunofluorescence staining revealed that *Ythdc2* cKO pachytene spermatocytes express and localize meiotic markers similar to control spermatocytes. At P16, *Ythdc2* cKO tubules were filled with pachytene spermatocytes that express high levels of the synaptonemal complex component SYCP3 (Figure 3A,B). Analysis of germ cell spreads revealed that SYCP3 properly localized on the chromosome axis in early (Figure 3E-H) and late (Figure 3I,J) spermatocytes from *Ythdc2* cKO males as in controls. *Ythdc2* cKO early prophase spermatocytes appeared to properly make double strand breaks (DSBs), as immunofluorescence staining of chromosome spreads showed γH2AX foci along the chromosomes in leptotene (Figure 3E,F) and zygotene (Figure 3G,H) spermatocytes. In pachytene spermatocytes, γH2AX became restricted to the sex vesicle containing the X and Y chromosomes in *Ythdc2* cKO males, similar to the control (Figure 3I,J). Analysis of the dynamics of RAD51 expression in chromosome spreads revealed that RAD51 foci were present at the zygotene stage (Figure 3-figure supplement 1A,B) then decreased in number in *Ythdc2* cKO pachytene spermatocytes similar to control (Figure 3-figure supplement 1C-F), suggesting that the double strand breaks were being properly repaired.

While pachytene spermatocytes appeared to have normal morphology and properly express meiotic markers at P16, the *Ythdc2* cKO germ cells failed to advance past the late pachytene stage and quickly underwent apoptosis, suggesting that YTHDC2 is required for late spermatocytes to complete meiotic prophase. Analysis of tubule cross-sections and cell spreads showed that while control tubules contained diplotene spermatocytes by P18 (Figure 2M and 3K), *Ythdc2* cKO male germ cells progressed to the diplotene stage very rarely (one diplotene cell in > 500 tubule cross-sections). Rather, primarily late pachytene spermatocytes were present in the P18 *Ythdc2* cKO tubules (Figure 2M and 3L). TUNEL staining showed a slight increase in the number of TUNEL-positive cells in *Ythdc2* cKO testes compared to controls starting at P16 (Figure 3N vs. M, quantified in Q). However, the number of TUNEL-positive cells per tubule cross-section greatly increased at P18 in *Ythdc2* cKO tubules compared to controls (Figure 3P vs. O, quantified in Q), with some tubules filled with TUNEL-positive spermatocytes in the *Ythdc2* cKO (Figure 3P, white arrow).

In addition to the inducible *Ythdc2* knockout model, *Ythdc2*^*flox/∆*^ conditional mice were also crossed to mice carrying the *Spo11-Cre* transgene, which expresses CRE recombinase in spermatocytes that have initiated meiosis (Lyndaker et al., 2013). Consistent with the findings from the inducible *UBC-Cre-ERT2; Ythdc2*^*flox/∆*^ mice, examination of P18 control and *Spo11-Cre; Ythdc2*^*flox/∆*^ testis tubules revealed that YTHDC2 was required to complete meiotic prophase. *Spo11-Cre; Ythdc2*^*flox/∆*^ germ cells reached the pachytene stage of meiotic prophase, as scored by immunofluorescence staining for γH2AX, which was restricted to the sex body as in control pachytene spermatocytes (Figure 3-figure supplement 2C,D). However, the spermatocytes then quickly started to die, leading to some tubules lacking late spermatocytes in P18 *Spo11-Cre; Ythdc2*^*flox/∆*^ testes (Figure 3-figure supplement 2B). Furthermore, analysis of histological sections from adult testis revealed that while the *Spo11-Cre; Ythdc2*^*flox/∆*^ model was not as penetrant as the inducible system, a large proportion of tubules lacked late-stage germ cells (Figure 3-figure supplement 2F vs. E), and mature sperm were rarely seen in cross-sections of the epididymis (Figure 3-figure supplement 2H vs. G). Taken together, our data from analysis of both the cell-stage specific and the inducible *Ythdc2* knockout mice revealed that in addition to its early role at the mitotic-to-meiotic transition, YTHDC2 has a distinct, later role in meiotic prophase regulating the pachytene-to-diplotene transition.

### YTHDC2 interacts with several other proteins involved in RNA regulation.

**Figure 4.**
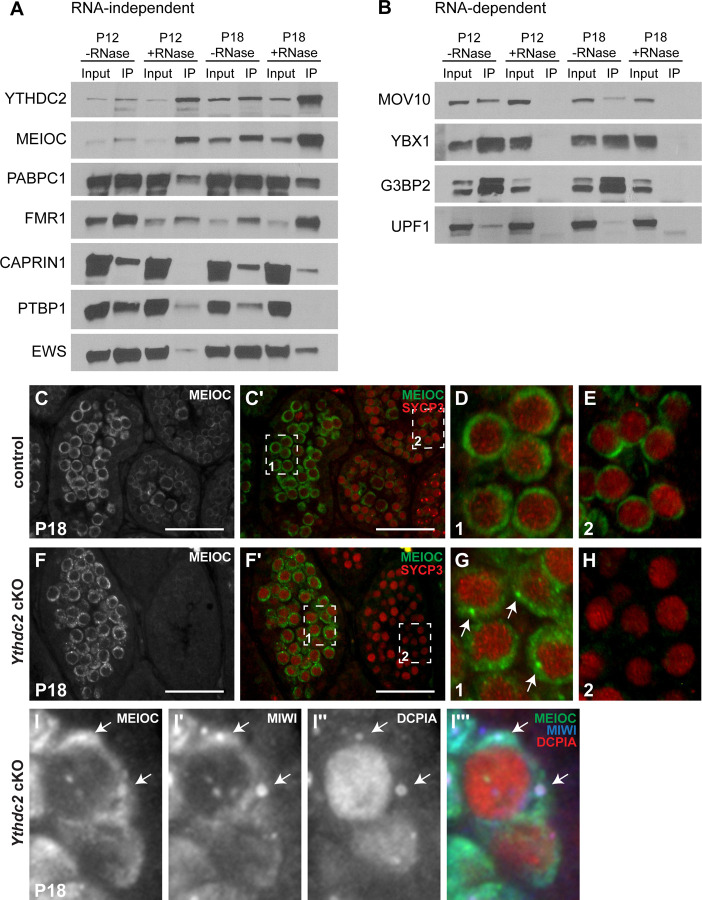
YTHDC2 interacts with several other proteins involved in RNA regulation. (A, B) Immunoprecipitation with α-YTHDC2 from P12 and P18 wild-type mouse testis extracts containing either an RNase Inhibitor (-RNase) or RNase A (+RNase). Western blots probed with α-YTHDC2, α-MEIOC, α-PABPC1, α-FMR1, α-CAPRIN1, α-PTBP1, α-EWS, α-MOV10, α-YBX1, α-G3BP2 and α-UPF1. (C-H) Immunofluorescence images of testis cross-sections from P18 (C-E) control (*+/+; Ythdc2*^*flox/∆*^) and (F-H) *Ythdc2* cKO (*UBC-CreERT2; Ythdc2*^*flox/∆*^) mice stained for MEIOC (green) and SYCP3 (red). Scale bars: 50 µm. (D, E) High-magnification images of (D) pachytene spermatocytes in boxed region 1 in panel Cʹ and (E) leptotene spermatocytes in boxed region 2 in panel Cʹ. (G, H) High-magnification image of (G) pachytene spermatocytes in boxed region 1 in panel Fʹ and (H) leptotene spermatocytes in boxed region 2 in panel Fʹ. Arrows: MEIOC protein in perinuclear puncta in pachytene spermatocytes. (I-Iʹʹʹ) High-magnification immunofluorescence images of spermatocytes from a P18 *Ythdc2* cKO mouse testis cross-section stained for MEIOC (green), MIWI (blue) and DCP1A (red). Arrows: puncta in the cytoplasm of pachytene spermatocytes. See also Figure 4 – source data 1 and Figure 4 – source data 2.

Consistent with its role in RNA regulation, YTHDC2 appears to interact with several other proteins involved in RNA biology, including RNA stability, RNA localization and translation. Immunoprecipitation of YTHDC2 from P12 and P18 wild-type testes followed by mass spectrometry revealed several novel candidate protein interacting partners for YTHDC2. As YTHDC2 is an RNA-binding protein, we validated candidate interacting partners by performing co-immunoprecipitation experiments in the presence and absence of RNase to uncover which proteins interact with YTHDC2 independently of RNA. Multiple proteins co-immunoprecipitated with YTHDC2 from RNase-treated testis extracts including the previously known interacting protein MEIOC as well as several additional proteins, including PABPC1, FMR1, CAPRIN1, PTBP1 and EWS (Figure 4A). MEIOC, PABPC1 and FMR1 showed particularly strong binding to YTHDC2 at P18 independent of RNA, while the hnRNP PTBP1 appeared to co-immunoprecipitate with YTHDC2 at low levels in the presence of RNase at P12. In addition to RNA-independent protein interactors, we identified three additional proteins that immunoprecipitated with YTHDC2 only in the presence of RNA, including MOV10, YBX1, G3BP2 and UPF1 (Figure 4B). Immunofluorescence staining of YTHDC2 and the protein binding partners in wild-type testes showed that the interactors were all highly expressed in the cytoplasm at specific spermatocyte stages. Some of the interactors were expressed at high levels primarily in early leptotene and zygotene spermatocytes, including PTBP1, MOV10 and G3BP2, while other interactors were highly expressed in pachytene spermatocytes, including PABPC1, CAPRIN1, YBX1 and UPF1 (Table 1).

MEIOC protein strongly co-immunoprecipitated with YTHDC2 from both P12 and P18 testes in the absence of RNA. Immunofluorescence staining of MEIOC and SYCP3 in P18 testes revealed that control and *Ythdc2* cKO tubules contained both pachytene spermatocytes (Figure 4Cʹ and Fʹ, box 1) as well as leptotene spermatocytes from the second wave of spermatogenesis (Figure 4Cʹ and Fʹ, box 2). In control testes, MEIOC was expressed in both pachytene (Figure 4D) and leptotene (Figure 4E) spermatocytes as previously shown (Abby et al., 2016; Soh et al., 2017). However, while MEIOC was present in the P18 *Ythdc2* cKO pachytene spermatocytes (Figure 4G), MEIOC was not detected by immunofluorescence in the *Ythdc2* cKO leptotene spermatocytes from the second wave (Figure 4H), suggesting YTHDC2 may promote the stability of MEIOC in early spermatocytes.

While MEIOC protein was present throughout the cytoplasm of *Ythdc2* cKO spermatocytes, it also appeared to concentrate in perinuclear puncta (Figure 4G, white arrows). In control spermatocytes, MEIOC protein was highly expressed throughout the cytoplasm and did not appear to accumulate as strongly in perinuclear puncta (Figure 4D). We have previously shown that YTHDC2 protein localizes to RNA granules in spermatocytes (Bailey et al., 2017). Co-staining with antibodies against two RNA granule components, the decapping enzyme DCP1A and the mouse PIWI family protein MIWI, revealed that many of the MEIOC-positive cytoplasmic puncta present in *Ythdc2* cKO spermatocytes also contained DCP1A and MIWI (Figure 4I-Iʹʹʹ, white arrows).

### Early transcript changes following knockout of *Ythdc2* in spermatocytes.

**Figure 5.**
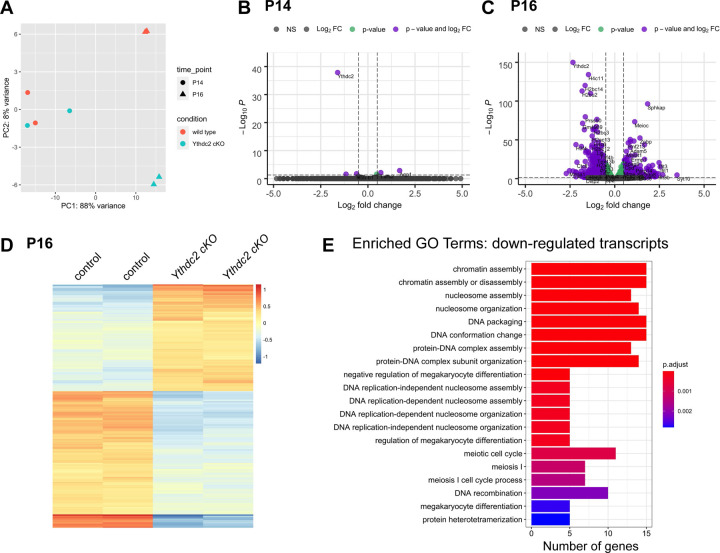
Early transcript changes following knockout of Ythdc2 in spermatocytes. (A) Principal component analysis (PCA) of the P14 and P16 control (*+/+; Ythdc2*^*flox/∆*^) and *Ythdc2* cKO (*UBC-CreERT2; Ythdc2*^*flox/∆*^) replicates. Principal component (PC) 1 splits the samples by time point and component 2 splits the samples by genotype. (B, C) Volcano plots representing significantly differentially expressed genes in *Ythdc2* cKO testes relative to control at (B) P14 and (C) P16. Significant differentially expressed genes with cutoffs: Log2FC > 1 or < −1 and p-value < 0.05 are represented in purple, differentially expressed genes with cutoff: p-value < 0.05 are represented in green and non differentially expressed genes are represented in grey. (D) Heatmap representing 294 transcripts differentially expressed in P16 *Ythdc2* cKO testes compared to controls. Biological replicates for both control and *Ythdc2* cKO are shown. Top: 129 transcripts higher in the *Ythdc2* cKO compared to the control (Log2FC > 1, p-value < 0.05). Bottom: 165 transcripts down in the *Ythdc2* cKO compared to the control (Log2FC < −1, p-value < 0.05). (E) The top 20 significant Biological Processes GO terms for the genes expressed at lower levels (Log2FC < −1, p-value < 0.05) in P16 *Ythdc2* cKO testes compared to control testes. See also Figure 5 – source data 1.

Comparison of RNA expression in *Ythdc2* cKO and control testes by RNA sequencing revealed very early changes in gene expression upon conditional knockout of *Ythdc2* in late spermatocytes. To observe early changes in gene expression following *Ythdc2* knockout that occur before the germ cells begin to die, we performed bulk RNA sequencing from control and *Ythdc2* cKO testes at P14 and P16 following two days of tamoxifen injections at P12 and P13. Principle component analysis (PCA) revealed that RNA expression in testes from control and *Ythdc2* cKO mice were similar at P14 (Figure 5A). At this time point, only one day post the second tamoxifen injection, levels of YTHDC2 protein detected by immunofluorescence staining of testis tubules were decreasing but YTHDC2 protein was still detected in some tubules (Figure 1F and 1O). While *Ythdc2* RNA expression was significantly down-regulated in the *Ythdc2* cKO testes compared to controls at P14, there were only five other mRNAs that showed significant changes in expression between control and *Ythdc2* cKO testes (*Aqp1*, *Lars2*, *Hbb-b1*, *Ndor1* and *Meioc*) (Figure 5B). At P16, the *Ythdc2* cKO replicates clustered, and the control P16 replicates clustered with each other but the two genotypes were widely separated in gene expression space (Figure 5A).

Many RNAs were significantly different at P16 in the *Ythdc2* cKO testes compared to controls (Figure 5C). Further examination of the significant differentially expressed transcripts in P16 *Ythdc2* cKO testes compared to controls (p < 0.05, Log2FC ≥ 1 or ≤ −1, 294 total genes) (Figure 5 – source data 1) revealed that nearly half (129/294) of the differentially expressed RNAs were up-regulated in the *Ythdc2* cKO testes compared to controls (Figure 5D). GO-term analysis of the 129 RNAs up-regulated in *Ythdc2* cKO P16 testes compared to controls showed enrichment for RNAs associated with viral response and response to interferon-beta, consistent with the initiation of programed cell death characteristic of late spermatocytes in the *Ythdc2* cKO. Interestingly, we observed a much larger number of RNAs up-regulated in the conditional *Ythdc2* knockout (n = 129 up-regulated genes) compared to the number of RNAs up-regulated in the P12 *Ythdc2* null mutant RNA-seq dataset (n = 19 up-regulated genes) (Bailey et al., 2017), suggesting that YTHDC2 may act in mid to late spermatocytes to target a number of RNAs for degradation.

In addition, 165 RNAs were down-regulated in P16 *Ythdc2* cKO testes compared to controls. GO-term analysis revealed that the most down-regulated RNAs in *Ythdc2* cKO versus controls were enriched for RNAs associated with chromatin assembly/organization and meiosis (Figure 5E). Consistent with immunofluorescence experiments revealing proper protein expression of the meiotic markers SYCP3, γH2AX and RAD51 in P16 *Ythdc2* cKO spermatocytes, expression of many meiotic transcripts was not significantly affected in P16 *Ythdc2* cKO mice compared to controls. However, there was a subset of meiotic transcripts (*Rec114*, *M1ap*, *Msh5*, *Prdm9*, *Zcwpw1*, *Mei4*, *Ccnb1ip1*, *Stra8*, *Rad21l* and *Mei1*) that were significantly down-regulated in *Ythdc2* cKO testes compared to controls.

Comparison of our RNA-seq data at P16 to two recently published CLIP-seq datasets from P15 testes (Li et al., 2022) and adult testes (Saito et al., 2022) revealed that many of the top transcripts differentially expressed in *Ythdc2* cKO testes compared to controls were directly bound by YTHDC2 (Figure 5 – source data 1). Overall, only 21% of the 249 differentially expressed transcripts were identified as YTHDC2 targets in either CLIP-seq dataset. However, further analysis revealed that 76% of the top 25 most significant differentially expressed transcripts were YTHDC2 targets, suggesting that the expression changes may be a direct consequence of *Ythdc2* knockout since YTHDC2 binds these transcripts. Some of the top transcripts present at higher levels in P16 *Ythdc2* cKO testes were directly bound by YTHDC2, including *Meioc* RNA, suggesting that the YTHDC2/MEIOC protein binding complex may help regulate their own levels of expression. In addition, many of the top transcripts present at lower levels in *Ythdc2* cKO testes were also directly bound by YTHDC2, including many of the highly down-regulated histones (*H4c11*, *H4c12*, *H2bc14* and *H2bu2*) (Figure 5C), suggesting that YTHDC2 may stabilize these RNAs.

## DISCUSSION

The RNA-binding protein YTHDC2 plays two distinct roles in early versus late meiotic prophase. Germ cells in *Ythdc2* null mutant testes attempt to enter meiosis but fail to properly shut down the mitotic program, leading to an abnormal, mitosis-like division and apoptosis. Here we show that YTHDC2 has a second, critical role in pachytene spermatocytes, where it is required for meiotic progression during the late pachytene stage. Upon conditional knockout of *Ythdc2* in early spermatocytes at P12 during the first wave of spermatogenesis, germs cells properly expressed and localized multiple meiotic markers and, unlike *Ythdc2* null mutant leptotene spermatocytes, they did not express CYCLINA2 or undergo abnormal chromatin condensation, indicating that the cells committed to meiosis and successfully progressed to the pachytene stage. However, changes in gene expression were quickly observed by P16, and the *Ythdc2* cKO spermatocytes failed to complete the pachytene-to-diplotene transition and instead underwent apoptosis.

Because we induced *Ythdc2* knockout at P12 and P13 during the first wave of spermatogenesis and analyzed testes starting one day post two tamoxifen injections, our experimental setup did not have later germ cell stages, allowing us to clearly observe the initial defects that occur upon knockout of *Ythdc2* as the germ cells proceed synchronously through meiotic prophase. Our studies agree with observations made by Liu and colleagues, who analyzed the effects of conditional knockout of *Ythdc2* in adult testis (Liu et al., 2021), but our studies are more focused on the initial, likely more direct effects of loss of YTHDC2 function in spermatocytes. Because adult testes have germ cells at various stages of development, Liu et al. observed a mixture of both the early mitotic-to-meiotic phenotype as well as a later phenotype during late pachytene. Also, as Liu et al. administered tamoxifen in adult mice for five consecutive days and analyzed the mice starting at two days post tamoxifen, the adult *Ythdc2* cKO testes contained round and elongating spermatids in certain tubule stages likely from the spermatogenic waves before tamoxifen introduction.

YTHDC2 contains several RNA-binding domains, including a R3H domain, RNA helicase core module (DEXDc and HELICc motifs) and an OB fold. The RNA helicase domain appears to be required for the function of YTHDC2 in late spermatogenesis, as a point mutation in the YTHDC2 ATPase motif blocked meiotic prophase progress but not meiotic entry (Li et al., 2022; Saito et al., 2022). YTHDC2 has been shown to have ATP-dependent, 3ʹ → 5ʹ RNA unwinding activity (Jain et al., 2018; Wojtas et al., 2017). The YTHDC2 ATPase point mutant phenotype appears similar to the later phenotype we and Liu et al. observed in *Ythdc2* cKO mice rather than the earlier, mitosis-to-meiosis phenotype seen in *Ythdc2* null mutants, indicating that YTHDC2’s helicase activity is required for is function in late pachytene spermatocytes but not in early spermatocytes. YTHDC2 also contains an YT521-B homology (YTH) domain that is not present in *Drosophila Bgcn*. The YTH domain of all five YTH family members can specifically recognize the N^6^-methyladenosine (m^6^A) RNA modification (Xu et al., 2015). YTHDC2 has been shown to preferentially bind m^6^A modified RNAs (Hsu et al., 2017; Kretschmer et al., 2018; Wojtas et al., 2017), although with much weaker affinity than other YTH domain proteins (Xu et al., 2015). However, binding of the m^6^A modification does not appear be critical for YTHDC2 function in the testis, as disrupting the YTH domain did not lead to any defects in spermatogenesis (Li et al., 2022; Saito et al., 2022).

Taken together, the data from our lab and others suggest that YTHDC2 may be playing two different functional roles in early versus late meiotic prophase. We and others have shown that a primary initial role of YTHDC2 in male germ cells entering meiotic prophase is to destabilize mitotic transcripts, including *Cyclin A2* (Bailey et al., 2017; Hsu et al., 2017; Soh et al., 2017; Wojtas et al., 2017). We now show here, using a conditional knockout strategy to bypass the first functional requirement, that YTHDC2 plays a key second role in pachytene spermatocytes.

Our RNA-seq data in P16 *Ythdc2* cKO testes identified the earliest transcript changes upon *Ythdc2* knockout in spermatocytes. Interestingly, a large percentage (76%) of the top 25 most significant differentially expressed genes were direct targets of YTHDC2 based on analysis by CLIP (Li et al., 2022; Saito et al., 2022). Of the 19 YTHDC2 target mRNAs in this group, five were detected at higher levels in P16 *Ythdc2* cKO testes compared to controls (*Sphkap*, *Meioc*, *Zpbp, C1galt1* and *Slc16a7*). The remaining 14 target mRNAs were detected at lower levels in P16 *Ythdc2* cKO testes compared to controls, suggesting that these key dysregulated transcripts may be stabilized directly by YTHDC2 during meiotic prophase.

While post-transcriptional regulation through action of YTHDC2 may alter RNA abundance for some YTHDC2 targets, we found that the changes in transcript levels by RNA-sequencing in both the *Ythdc2* null mutant (Bailey et al., 2017) and the *Ythdc2* cKO were often quantitatively small, and many direct YTHDC2 RNA targets identified by CLIP in both early (Saito et al., 2022) and late (Li et al., 2022; Saito et al., 2022) spermatocytes showed no changes in RNA expression levels at the time points we assessed, suggesting that binding of YTHDC2 could affect other processes, such as promoting or repressing translation or regulating localization or storage of the target RNAs. Previous studies have suggested that YTHDC2 could promote translation of certain RNA targets, as YTHDC2 interacts with the small ribosomal subunit (Kretschmer et al., 2018) and binding of YTHDC2 to transcript coding sequences (CDS) may help resolve mRNA secondary structures and promote translation *in vitro* (Mao et al., 2019). While a recent study performed ribosome profiling on P8 and P10 testes and found that loss of *Ythdc2* did not substantially alter the translation of YTHDC2 targets (Saito et al., 2022), indicating that YTHDC2 likely does not regulate translation of its targets at the mitotic to meiotic switch, it remains to be determined whether YTHDC2 may act through translational regulation in late spermatocytes. YTHDC2 CLIP experiments from P8, P10 and adult testes suggest that the RNA targets may vary across germ cell stages (Saito et al., 2022). Therefore, YTHDC2 action on target RNAs could be different depending on factors such as where on the transcript YTHDC2 binds (CDS versus 3ʹUTR) (Li et al., 2022; Mao et al., 2019; Saito et al., 2022) or what other YTHDC2-binding partners are present when the target RNA is bound.

Several studies have previously determined that YTHDC2 is part of a protein complex containing MEIOC (Abby et al., 2016; Bailey et al., 2017; Soh et al., 2017), the RNA-binding protein RMB46 (Li et al., 2022; Qian et al., 2022) as well as the exoribonuclease XRN1 (Kretschmer et al., 2018; Li et al., 2022; Wojtas et al., 2017). To elucidate the mechanism(s) of action of YTHDC2 during early and late meiotic prophase, we performed YTHDC2 immunoprecipitation experiments in P12 and P18 testis extracts, which revealed both RNA-independent and RNA-dependent YTHDC2 interacting partners in early and late spermatocytes. All the YTHDC2-bound interacting proteins we detected have known roles in regulating RNA biology, consistent with the proposed functional role of YTHDC2 in regulating gene expression through regulation of target RNAs. YTHDC2 may associate with specific protein complexes at different cell stages, leading to diverse, stage-specific molecular outcomes on RNA targets. Similar RBM46 IP Mass-spec experiments performed in P12 and P21 mouse testes revealed a number of RBM46-associated proteins, several of which were also present in the YTHDC2 protein complexes we identified, including MEIOC, PABPC1, MOV10 and UPF1 (Qian et al., 2022).

In our previous study examining the subcellular localization of YTHDC2, we showed that while YTHDC2 is distributed throughout the cytoplasm, it also concentrated in perinuclear puncta that co-expressed the P-body marker DCP1A (Bailey et al., 2017). Here we show that MEIOC protein strongly accumulated in cytoplasmic puncta that co-expressed the granule markers DCP1A and MIWI in *Ythdc2* cKO testis tubules. Interestingly, MEIOC protein did not appear as enriched in cytoplasmic puncta in control spermatocytes. One explanation for why knockout of *Ythdc2* in spermatocytes leads to increased MEIOC in puncta could be that YTHDC2 is directly acting to move MEIOC out of the granules in pachytene spermatocytes. Alternatively, MEIOC accumulation in granules could be an indirect effect of YTHDC2 deficiency. For example, other MEIOC binding partners could facilitate increased puncta localization in the absence of YTHDC2. In addition to YTHDC2 and MEIOC, all of the additional protein interacting partners identified in this study have also been shown to be components of P-bodies or other types of RNP granules. Taken together, these data suggest that the YTHDC2 complex may recruit target RNAs into granules where they are degraded, translationally silenced or stored depending on the components of the granule, and/or return target RNAs from granules to the cytoplasm where they can be translated.

The conserved mouse proteins YTHDC2, MEIOC and RBM46 function together to regulate proper entry and execution of the mitosis-to-meiosis transition. All three proteins play a critical role at the switch, as germ cells null mutant for any of the three proteins attempt to enter the meiotic program but fail to shut off the mitotic program. As our current data reveal that YTHDC2 has an additional role in late spermatocytes, it would be interesting to examine whether MEIOC and RBM46 also function during late meiotic prophase, or if YTHDC2 may instead work with other proteins to facilitate the transition from late pachytene to diplotene spermatocytes.

## MATERIALS AND METHODS

### Mice

*Ythdc2* conditional knockout mice were generated according to the scheme depicted in Figure 1-figure supplement 1. *Ythdc2*^*tp/tp*^ mice (Bailey et al., 2017) were crossed to C57BL/6N-Tg(CAG-Flpo)1Afst/Mmucd (RRID:MMRRC_036512-UCD) mice which removed the gene-trap cassette by Flp recombinase and reverted the mutation to wild type, with LoxP sites flanking exons 6 and 7 (*Ythdc2*^*flox*^). Removal of the gene-trap cassette was confirmed by PCR using the following primers: *Ythdc2* forward 5ʹarm: 5ʹ-CTGAACATGTCTTATCCACAGTGC-3ʹ, *Ythdc2* reverse 3ʹarm: 5ʹ-CATCATCAAGAAGGTTACAACAGGC-3ʹ, Neo forward: 5ʹ-CAGCGCATCGCCTTCTATCGCC-3ʹ. The *Ythdc2*^*flox*^ mice were then crossed to mice carrying the *Ythdc2* null allele (*Ythdc2*^*+/∆*^) (Bailey et al., 2017) to generate *Ythdc2*^*flox/∆*^ mice. *Ythdc2*^*flox/∆*^ mice were crossed to either the inducible Cre strain *B6.Cg-Ndor1*^*Tg(UBC-cre/ERT2)1Ejb*^*/1J* (RRID:IMSR_JAX:007001) (Ruzankina et al., 2007) or to *Tg(Spo11-cre)1Rsw/PecoJ* (RRID:IMSR_JAX:032646) (Lyndaker et al., 2013). Mice were genotyped by PCR using the following primers: *Cre* forward: 5ʹ-TGGGCGGCATGGTGCAAGTT −3ʹ, *Cre* reverse: 5ʹ-CGGTGCTAACCAGCGTTTTC −3ʹ, *Ythdc2* forward: 5ʹ-CGAGTGCTGCCTTGGATGTGAACC −3ʹ, *Ythdc2* reverse: 5ʹ-GGATTTTGACAGCCTTGAGCCTGGG −3ʹ, targeting cassette (*LacZ*) forward: 5ʹ-GAATTATGGCCCACACCAGTGGCG −3ʹ. All experiments were approved by the Stanford University Animal Care and Use Committee and performed according to NIH guidelines.

### *Ythdc2* knockout with Tamoxifen

Tamoxifen (10 mg/ml, Cayman Chemical) was dissolved in corn oil by incubating at 55°C for 40 min with constant rotation and then 0.1 mg/g body weight was injected intraperitoneally using a 27G needle into *UBC-Cre*^*ERT2*^*;Ythdc2*^*flox/∆*^ mice at P12 and P13 at the same time each day. *Ythdc2*^*flox/∆*^ male mice lacking *UBC-Cre*^*ERT2*^ were injected with tamoxifen and used as controls.

### Histology

Mouse testes were fixed overnight at room temperature (RT) in bouins fixative and embedded in paraffin. Paraffin-embedded samples were cut to 5–6 μm sections and stained with periodic acid-Schiff (PAS).

### Immunofluorescence

For immunofluorescence staining, tissues were fixed with 4% paraformaldehyde overnight at 4°C, embedded in paraffin and cut to 5–6 μm thickness. After rehydrating in an ethanol gradient, heat-mediated antigen retrieval was performed on the sections using sodium citrate buffer (10 mM sodium citrate, 0.05% Tween-20, pH 6.0). Sections were then permeabilized with phosphate-buffered saline + 0.1% TritonX-100 (PBST) for 45 min at RT, followed by 1 hr incubation with blocking buffer (10% BSA in PBST) at RT. Sections were incubated with primary antibody overnight at 4°C and then incubated with Alexa Fluor-conjugated donkey secondary antibodies (1:400, Molecular Probes) at RT for 2 hrs and then mounted in VECTASHIELD medium with 4′−6-Diamidino-2-phenylindole (DAPI) (Vector Lab Inc.). When immunolabeling with antibodies from the same host species, following incubation with primary antibody overnight at 4°C, slides were washed 3 times in PBS, and then incubated with HRP anti-rabbit IgG (1:500 in 10% BSA in PBST) for 1 hr at RT. Following incubation, TSA plus cyanine 3 solution (dilute TSA-Cy3 1:250 in 100mM Boric Acid, pH8.5 containing 0.003% H_2_O_2_) (Akoya Biosciences) was added to the tissue sections and incubated for 10 min at RT. Slides were then washed twice in PBS and antigen retrieval using sodium citrate buffer was performed a second time. Sections were blocked and then incubated with primary and secondary antibodies as described above.

### Antibodies

Rabbit anti-YTHDC2 (1:500 or 1:2,000 when TSA used, A303–026A, (RRID:AB_10754785), Bethyl laboratories), mouse anti-SYCP3 (1:200, clone [Cor 10G11/7], (RRID:AB_10678841), Abcam), rabbit anti-SYCP3 (1:500, NB300–232, (RRID:AB_2087193), Novus Biologicals), rabbit anti-RAD51 (1:200 [N1C2], (RRID:AB_1951602), GeneTex), goat anti-VASA (1:200, AF2030, (RRID:AB_2277369), R&D Systems), mouse anti-phospho-Histone H2A.X (Ser139) (1:500 clone [JBW301], (RRID:AB_309864), Millipore), rabbit anti-CYCLIN A2 (1:250 clone [EPR17351], (RRID:AB_2890136), Abcam), rabbit anti-MEIOC (1:1,000 IF David Page Lab), mouse anti-DCP1A (1:250 clone [3G4], (RRID:AB_1843673), Sigma), goat anti-PIWIL1 (1:200, AF6548, (RRID:AB_10971944), Novus Biologicals). Terminal deoxynucleotidyl transferase dUTP nick end labeling (TUNEL) staining was done following manufacturer’s instruction (In Situ cell Death Detection Kit TMR red, Roche).

The following antibodies were used for immunoblot: rabbit anti-YTHDC2 (1:1,000, A303–026A, (RRID:AB_10754785), Bethyl laboratories), rabbit anti-MEIOC (1:2000 immunoblot, David Page Lab), goat anti-RENT1/UPF1 (1:2,000, A300–038A, (RRID:AB_2288326), Bethyl laboratories), rabbit anti-EWS (1:2,000, A300–417A, (RRID:AB_420957), Bethyl laboratories), rabbit anti-MOV10 (1:2,000, A301–571A, (RRID:AB_1040002), Bethyl laboratories), rabbit anti-G3BP2 (1:2,000, A302–040A, (RRID:AB_1576545), Bethyl laboratories), rabbit anti-YBX1 (1:2,000, A303–231A, (RRID:AB_10951283), Bethyl laboratories), mouse anti-PTBP1 (1:2,000, Thermo), rabbit anti-PABPC1 (1:1,000, ab21060, (RRID:AB_777008, Abcam), rabbit anti-FMR1 (1:2,000, A305–200A, (RRID:AB_2631593), Bethyl laboratories), rabbit anti-CAPRIN1 (1:2,000, HPA018126, (RRID:AB_1849929), Sigma).

### Germ-cell spreads

Chromosome spreads from P16 and P18 testes were performed based on a previously published protocol (Peters et al., 1997). Briefly, testis tubules were incubated in a hypotonic extraction buffer pH 8.2 (30 mM Tris-HCL pH 8.0, 50 mM sucrose, 17 mM trisodium citrate dehydrate, 5 mM EDTA, 0.5 mM DTT, 0.5 mM PMSF) for 30 min. Testis tubules were then broken apart in 100 mM sucrose pH 8.2 and then spread on a slide dipped in fixation buffer pH 9.2 (1% PFA and 0.15% Triton X-100). Slides were incubated overnight in a humidified chamber at RT and air-dried. Slides were washed twice in 0.4% Photoflo (Kodak) and air-dried.

### Co-immunoprecipitation from testes

Anti-YTHDC2 (A303–025A, Bethyl laboratories) was conjugated to Protein A Dynabeads (Invitrogen). Dynabeads (50 μL/IP) were blocked briefly in 3% BSA in PBS + 0.1% tween 20 (PBST) and then incubated at 4°C overnight in lysis buffer (20 mM Tris-HCL, 135 mM NaCl, 10% glycerol, 1% Nonidet P-40, 5 mM EDTA) plus 2 μg YTHDC2 antibody. Beads were washed three times with 0.2 M triethanolamine, pH 8.2 for 5 min at RT, then incubated with dimethylprimelimidate (DMP, 5.4 mg DMP per mL 0.2 M triethanolamine, Sigma) for 30 min at RT. Beads were washed once with 50 mM Tris for 15 min, followed by three 5-min washes with PBST. Crosslinked beads can be stored overnight at 4°C or used immediately in the next step. To remove un-crosslinked antibody, beads were quickly washed twice with 100mM glycine, pH2.5, then blocked in 3% BSA PBST for 30 min followed by three quick PBST washes.

For immunoprecipitations from testis extracts, testes were dissected from P12 and P18 wild-type mice. Testis extracts were prepared by mechanically disrupting testes in lysis buffer (20 mM Tris-HCL, 135 mM NaCl, 10% glycerol, 1% Nonidet P-40, 5 mM EDTA, 1 mM PMSF, 1x Complete protease inhibitor, and either (A) 100 U/mL RNAse-OUT or (B) 50 µg/mL RNAse A). Extracts were incubated in lysis buffer for 20 min at 4°C, spun for 20 min, and then precleared with uncoupled, 3% BSA-blocked Protein A Dynabeads for 1 hr at 4°C. Testis extracts were then added to the crosslinked YTHDC2 antibody - Protein A Dynabeads and incubated for 3 hrs at 4°C while rotating. Beads were washed three times in lysis buffer for 5 min each wash, and incubated at 70°C for 30 min in elution buffer (1% SDS, 10 mM EDTA, 50 mM Tris pH 8.0, 1 mM PMSF, 1x Complete protease inhibitor) with frequent mixing. Samples were resolved on a 10% SDS-PAGE gel (Bio-Rad) and transferred onto polyvinylidene fluoride (PVDF) membranes. Blots were then incubated in primary antibodies overnight at 4°C and then IgG HRP secondary antibodies (1:10,000) for 2 hrs at RT and then developed using western lightning ECL detection reagent.

### Mass-Spectometry

Testes were dissected from P12 and P18 wild-type and *Ythdc2* mutant mice. YTHDC2 immunoprecipitations were performed as described above with slight modifications: the uncoupled, preclear Protein A Dynabeads were washed with PBST to remove unbound BSA prior to adding to the testis lysate, and the crosslinked YTHDC2 antibody - Protein A Dynabeads were not blocked with 3% BSA following DMP crosslinking. Testis extracts were precleared with uncoupled beads for 3 hrs at 4°C followed by incubation with the crosslinked YTHDC2 antibody - Protein A Dynabeads for 3 hrs at 4°C with continuous rotation. Beads were washed three times in lysis buffer for 10 min each wash, and then incubated in elution buffer as previously described above. Samples were resolved on a 10% SDS-PAGE gel (Bio-Rad). The samples were allowed to run about 1 cm into the gel and the gel was then fixed for 1 hr in fixing solution (45:45:10 / water:methanol:acetic acid) with gentle agitation. The samples were cut out of the gel and stored at 4°C until being processed at the Stanford University Mass Spectrometry Facility.

### RNA-seq from testes

Testes were collected from P14 and P16 *UBC-Cre*^*ERT2*^*;Ythdc2*^*flox/∆*^ and *Ythdc2*^*flox/∆*^ control mice following tamoxifen injections at P12 and P13. One testis was snap frozen and the other was fixed in 4% formaldehyde to confirm *Ythdc2* knockout. Total RNA was isolated from frozen testes using the RNeasy Plus Mini Kit (Qiagen), according to manufacturer’s instructions. Ribosomal RNAs were depleted from 1 µg of total RNA using TruSeq Stranded Total RNA kit Ribo-Zero components (Illumina) according to the manufacturer’s protocol. Ribosomal-depleted RNA was cleaned up using Agencourt RNAClean XP Beads (Beckman Coulter) and efficiency of rRNA depletion was confirmed by bioanalyzer. Library preparation for high-throughput sequencing was performed on 100 ng of rRNA depleted RNA using the NEBNext Ultra II Directional RNA Library Prep Kit for Illumina (New England Biolabs). Sequencing was done with a NextSeq 500/550 High Output Kit v2.5 (150 Cycles), with eight libraries pooled. Approximately 55 million to 66 million reads were obtained per replicate and each condition had two biological replicates.

### RNA expression analysis

Libraries were separated by barcode and adapters and low-quality bases were trimmed using trimGalore (Martin, 2011). Reads were mapped to the mouse genome (GRCm39/mm39) using STAR (RRID:SCR_004463) (Dobin et al., 2013). Reads for each transcript were extracted using HTSeq (RRID:SCR_005514) (Anders et al., 2015). Differential gene expression was calculated using DESeq2 (RRID:SCR_015687) (Love et al., 2014).

### Statistical analysis

Biological replicates were performed for RNA-Seq experiments and samples for each biological replicate were collected from different animals. For phenotypic analysis, sample sizes (n) for each experiment are given in the respective figure legends. Sample sizes used were similar to what is generally utilized in the field. Each animal was considered a biological replicate. No samples were excluded from analysis. All bar graphs are presented as means ± SEM. Statistical differences between two groups were analyzed using unpaired two-tailed t tests; p<0.05 was considered statistically significant.

### Data availability

RNA-seq data have been deposited in the Gene Expression Omnibus (GEO) under accession number GSE222283.

## Supplementary Material

Supplement 1

## Figures and Tables

**Table 1.** Expression pattern of YTHDC2 protein binding partners in spermatocytes

Protein	Function	Protein expression pattern in spermatocytes by immunofluorescence
MEIOC	Regulation of RNA stability	Leptotene, zytotene and pachytene spermatocytes (cytoplasmic)
PABPC1	Regulation of RNA stability, translation initiation and RNA degradation.	Low in leptotene and zygotene / high in pachytene spermatocytes (cytoplasmic)
FMR1	Regulaton of RNA stability, translation elongation, RNA processing and RNA transport	Leptotene, zygotene and early pachytene spermatocytes (cytoplasmic)
CAPRIN1	Regulation of translation and RNA transport	Pachytene spermatocytes (cytoplasmic)
PTBP1	Regulation of alternative splicing (nucleus) and RNA localization, stability and translation (cytoplasm)	Leptotene and zygotene spermatocytes (cytoplasmic)
EWSR1	Transciption coregulator activity and RNA binding	Leptotene zytotene (cytoplasmic and nuclear), pachytene (nuclear)
MOV10	Regulation of RNA stability, translation, RNA cleavage/gene silencing by miRNA	Leptotene and zygotene spermatocytes (cytoplasmic)
YBX1	Regulation of RNA stability, RNA processing, translational repression and splicing	Pachytene spermatocytes (cytoplasm)
G3BP2	Scaffold protein, granule assembly, RNA transport	Leptotene and zygotene spermatocytes (cytoplasmic)
UPF1	Regulation of nonsense mediated mRNA decay, RNA destabilization	Pachytene spermatocyts (cytoplasmic)
