# Supplementary material for: YTHDC2 serves a distinct late role in spermatocytes during germ cell differentiation
Source: bioRxiv. 2023 Jan 23:2023.01.23.525146. Preprint. [Version 1] doi: 10.1101/2023.01.23.525146 (PMC9900820; doi:10.1101/2023.01.23.525146)
Supplement: Supplementary file 1 [file NIHPP2023.01.23.525146v1-supplement-1.pdf]

bioRxiv preprint doi: https://doi.org/10.1101/2023.01.23.525146; this version posted January 23, 2023. The copyright holder for this preprint (which was not certified by peer review) is the author/funder, who has granted bioRxiv a license to display the preprint in perpetuity. It is made available under aCC-BY 4.0 International license.

FIGURE SUPPLEMENTS

Figure 1 - figure supplement 1.

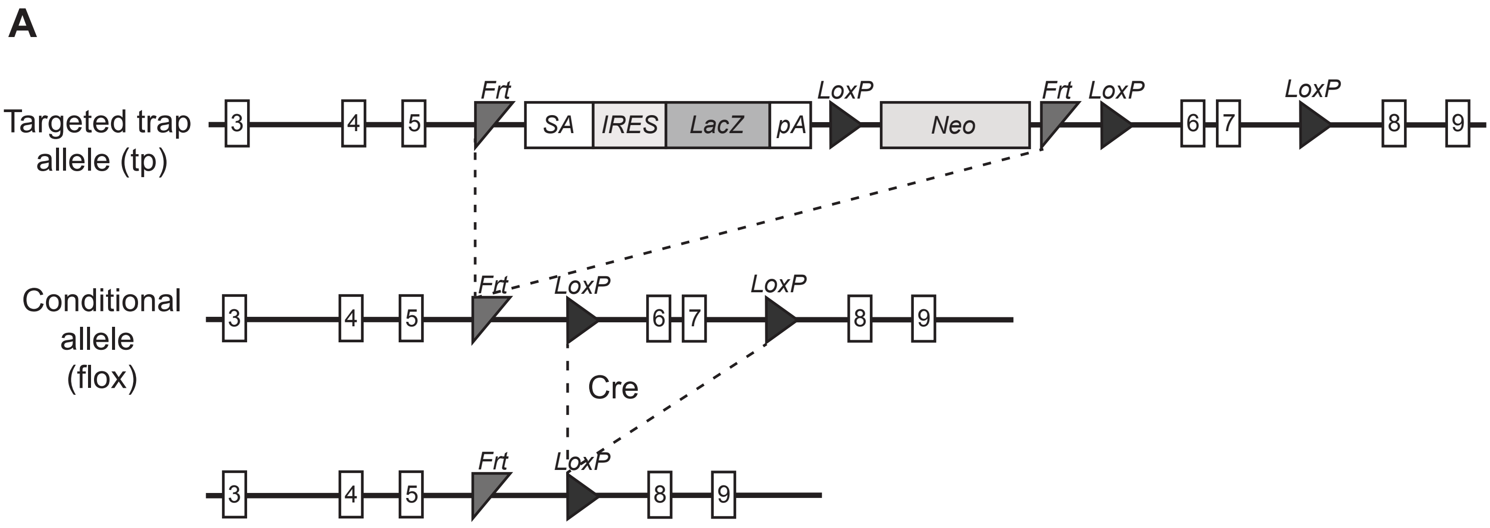

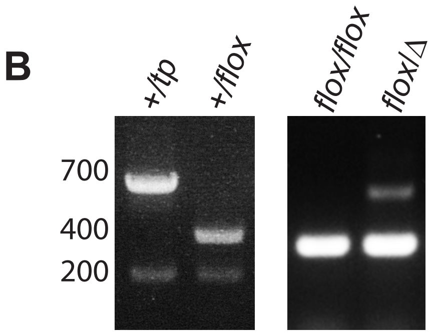

bioRxiv preprint doi: https://doi.org/10.1101/2023.01.23.525146; this version posted January 23, 2023. The copyright holder for this preprint (which was not certified by peer review) is the author/funder, who has granted bioRxiv a license to display the preprint in perpetuity. It is made available under aCC-BY 4.0 International license.

Figure 1 – figure supplement 1. Generation of the *Ythdc2* conditional knockout mouse.

(A) Diagram of the generation of the *Ythdc2* conditional allele (*flox*) from the targeted trap allele (*tp*) through excision of the LacZ reporter and the Neomycin cassette. Cre-mediated deletion of exons 6 and 7 occurs in the *Ythdc2* floxed mice upon exposure to Cre recombinase. SA, splice acceptor; IRES, internal ribosome entry site; LacZ, β-galactosidase gene; pA, polyadenylation sequence; LoxP, Cre recombinase recognition site; FRT, flippase recognition site. (B) Mouse genotyping by PCR analysis of DNA purified from mice heterozygous for the targeted trap allele (+/*tp*), heterozygous for the conditional allele (+/*flox*), homozygous for the conditional allele (*flox/flox*) and heterozygous for the conditional and null allele (*flox*/Δ).

bioRxiv preprint doi: https://doi.org/10.1101/2023.01.23.525146; this version posted January 23, 2023. The copyright holder for this preprint (which was not certified by peer review) is the author/funder, who has granted bioRxiv a license to display the preprint in perpetuity. It is made available under aCC-BY 4.0 International license.

Figure 3 - figure supplement 1.

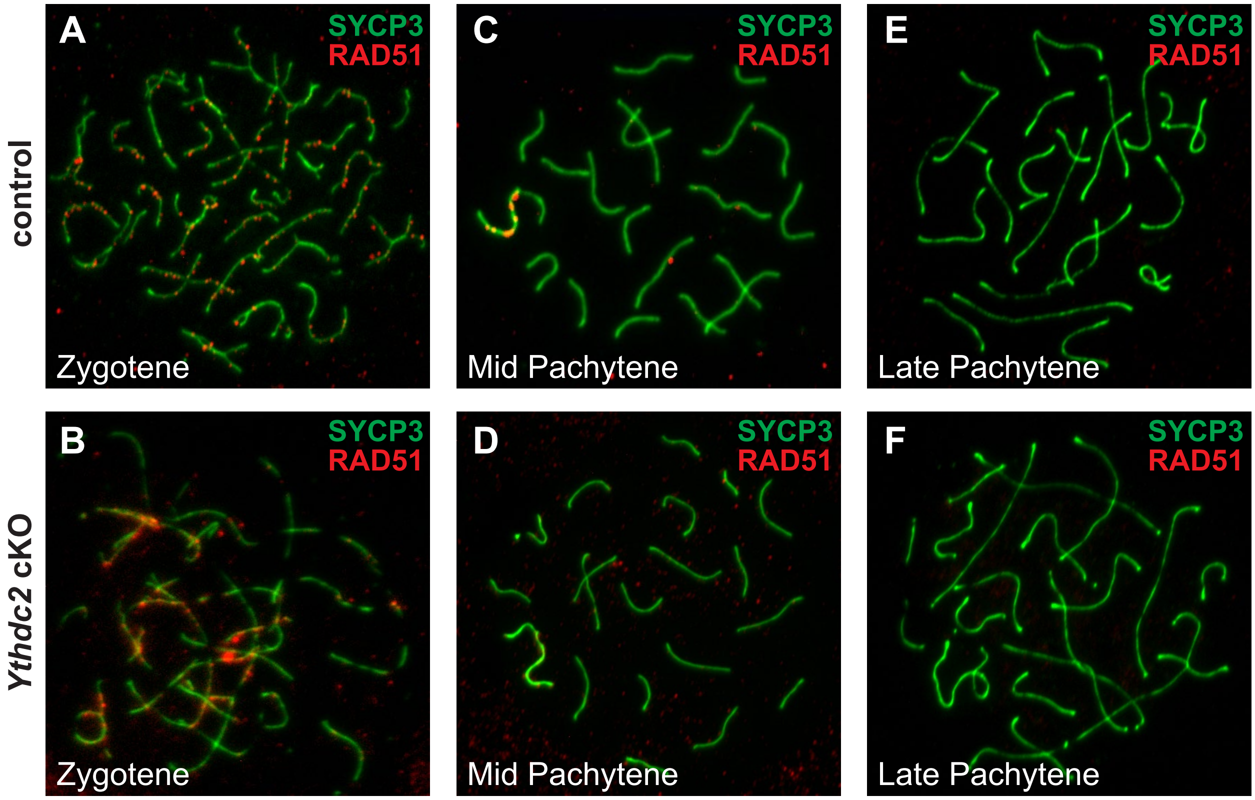

bioRxiv preprint doi: https://doi.org/10.1101/2023.01.23.525146; this version posted January 23, 2023. The copyright holder for this preprint (which was not certified by peer review) is the author/funder, who has granted bioRxiv a license to display the preprint in perpetuity. It is made available under aCC-BY 4.0 International license.

Figure 3 – figure supplement 1. Normal expression pattern of RAD51 in early and late spermatocytes. (A-F) Immunofluorescence images of germ cell spreads from P18 control (+/+*; Ythdc2*$^{flox/\Delta}$, top) and *Ythdc2* cKO (*UBC-CreERT2; Ythdc2*$^{flox/\Delta}$, bottom) mice stained for SYCP3 (green) and RAD51 (red).

bioRxiv preprint doi: https://doi.org/10.1101/2023.01.23.525146; this version posted January 23, 2023. The copyright holder for this preprint (which was not certified by peer review) is the author/funder, who has granted bioRxiv a license to display the preprint in perpetuity. It is made available under aCC-BY 4.0 International license.

Figure 3 - figure supplement 2.

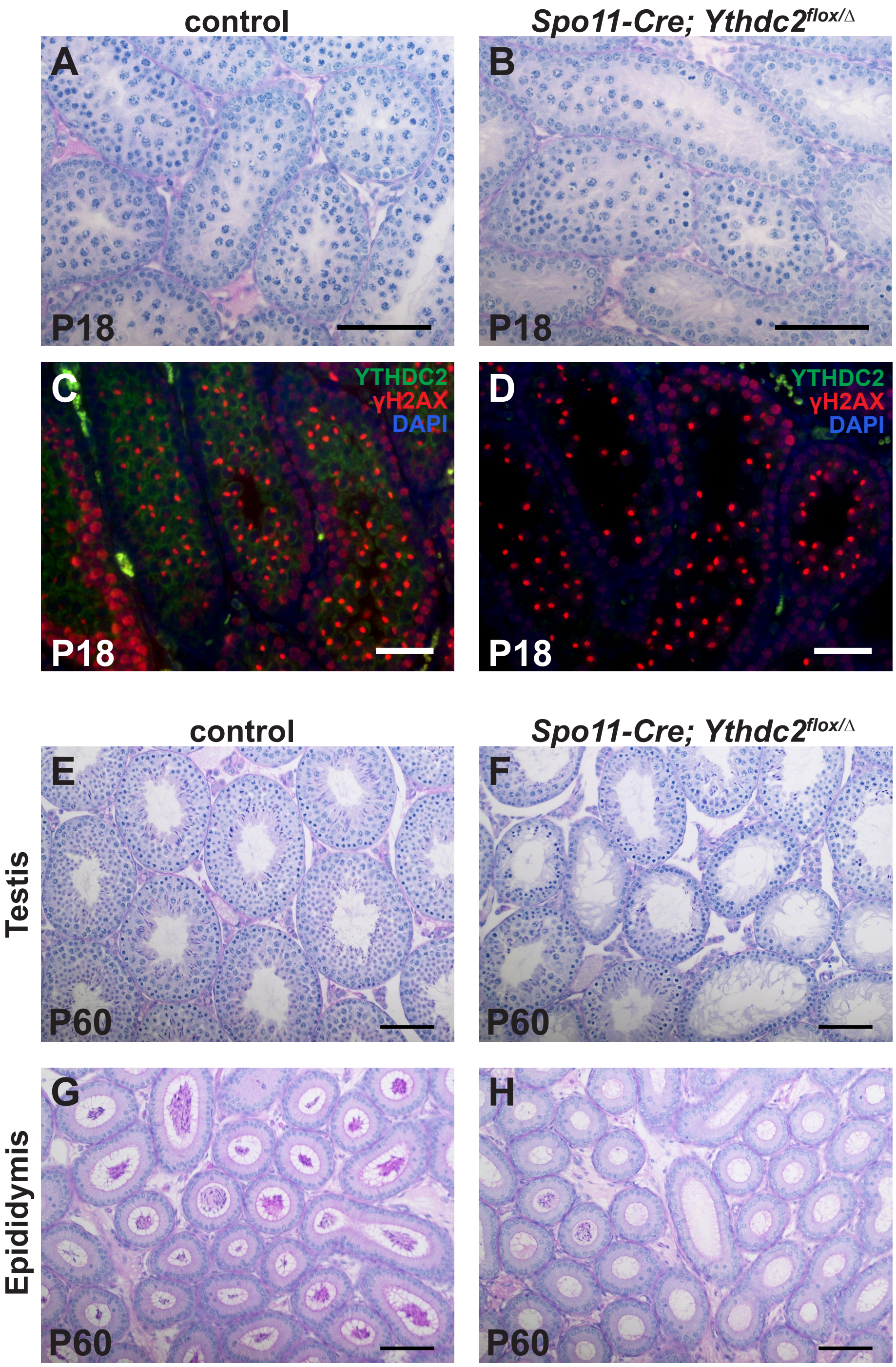

bioRxiv preprint doi: https://doi.org/10.1101/2023.01.23.525146; this version posted January 23, 2023. The copyright holder for this preprint (which was not certified by peer review) is the author/funder, who has granted bioRxiv a license to display the preprint in perpetuity. It is made available under aCC-BY 4.0 International license.

Figure 3 – figure supplement 2. Knocking out *Ythdc2* using the *Spo11-Cre* driver leads to a similar phenotype as the inducible driver strategy. (A, B) Cross-sections of mouse seminiferous tubules stained with periodic acid-Schiff (PAS) from P18 (A) control and (B) *Spo11-Cre; Ythdc2*$^{flox/\Delta}$ mice. (C, D) Immunofluorescence images of P18 (C) control and (D) *Spo11-Cre; Ythdc2*$^{flox/\Delta}$ mouse testis tubule cross-sections stained for YTHDC2 (green), γH2AX (red) and DAPI (blue). (E, F) Cross-sections of mouse seminiferous tubules stained with periodic acid-Schiff (PAS) from P60 (E) control and (F) *Spo11-Cre; Ythdc2*$^{flox/\Delta}$ mice. (G, H) Cross-sections of mouse epididymis stained with periodic acid-Schiff (PAS) from P60 (G) control and (H) *Spo11-Cre; Ythdc2*$^{flox/\Delta}$ mice. Scale bars: 50 μm.

bioRxiv preprint doi: https://doi.org/10.1101/2023.01.23.525146; this version posted January 23, 2023. The copyright holder for this preprint (which was not certified by peer review) is the author/funder, who has granted bioRxiv a license to display the preprint in perpetuity. It is made available under aCC-BY 4.0 International license.

LIST OF FIGURE SUPPLEMENTS AND SOURCE DATA FILES:

Figure 1 – figure supplement 1. Generation of the *Ythdc2* conditional knockout mouse.

Figure 3 – figure supplement 1. Normal expression pattern of RAD51 in early and late spermatocytes.

Figure 3 – figure supplement 2. Knocking out *Ythdc2* using the *Spo11-Cre* driver leads to a similar phenotype as the inducible driver strategy.

Figure 4 - source data 1. Original files for western blots in Figure 4A.

Figure 4 - source data 2. Original files for western blots in Figure 4B.

Figure 5 - source data 1. Differentially expressed genes in P16 *Ythdc2* cKO compared to control testes.
